# Comparative analysis of gut microbiota diversity in endangered, economical, and common freshwater mussels using 16S rRNA gene sequencing

**DOI:** 10.1002/ece3.6796

**Published:** 2020-10-13

**Authors:** Xiongjun Liu, Yanling Cao, Shan Ouyang, Xiaoping Wu

**Affiliations:** ^1^ School of Life Sciences Nanchang University Nanchang China; ^2^ School of Life Sciences Jiaying University Meizhou China

**Keywords:** conservation, diversity, freshwater mussels, gut microbiota

## Abstract

Freshwater mussels are both among the most diverse and endangered faunas worldwide. The gut microbiota of species plays a key role in nutrition and immunity, such as preventing it from pathogen invasion, synthesizing beneficial secondary metabolites, and contributing to the digestion of complex nutrients. Information on the gut microbiota could have significant implications for conservation biology, especially for threatened or endangered species. However, there is relatively little study into the gut microbiota of freshwater mussels. Here, the gut microbiota diversity was analyzed in endangered (*Solenaia carinata*), economical (*Sinohyriopsis cumingii*), and common (*Sinanodonta woodiana*) freshwater mussels using 16S rRNA gene sequencing. This study represents the first to compare the gut microbiota diversity of endangered, economical, and common Chinese freshwater mussels. The results showed that 13,535 OTUs were found in *S. carinata*, 12,985 OTUs in *S. cumingii,* and 9,365 OTUs in *S. woodiana*. The dominant phylum in *S. carinata* and *S. cumingii* was Fusobacteria, and was Firmicutes in *S. woodiana*. Alpha diversity indices indicated that *S. carinata* and *S. cumingii* had a higher abundance and diversity of gut microbiota than *S. woodiana*. The composition of gut microbiota was different among three freshwater mussels, but their composition variation was not significant. This study provides insight for the conservation of freshwater mussel biodiversity, which will not only help conserve these vulnerable groups but also, will offer wider benefits to freshwater ecosystems.

## Graphical abstract

This study represents the first to compare with the gut microbiota diversity in endangered, economical, and common Chinese freshwater mussels. The composition of gut microbiota among three freshwater mussels was different, but their composition variation was not significant. The composition of gut microbiota for three freshwater mussels was correlated with dissolved oxygen, turbidity, and chlorophyll‐a. This study provides valuable insight for the conservation of freshwater mussel biodiversity, which will not only help conserve these vulnerable groups but also, will offer wider benefits to freshwater ecosystems.

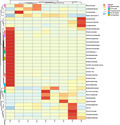

## 1 INTRODUCTION

The gastrointestinal tract of the animal is a complex micro‐ecosystem which contains numberous microbiota and approximately 1,000–5,000 species of microorganism dwell in it (Eckburg et al., 2005; Ley, Lozupone, Hamady, Knight, & Gordon, 2008). It is known that there is a "triangle" relationship of interaction and dependence existing among the microbiota, host, and the gastrointestinal tract environment (such as food, temperature, and pH), and jointly participate in digestion and absorption of nutrients and energy metabolism process (Flint, Scott, Louis, & Duncan, 2012). Many studies have elucidated that gut microbiota participate in host's nutrition metabolism and immune regulation. For example, the gut microbiota in vertebrates plays a key role in nutrition and immunity, such as preventing pathogen invasion, synthesizing beneficial secondary metabolites, and contributing to the digestion of complex nutrients (Nayak, 2010; Verschuere, Rombaut, Sorgeloos, & Verstraete, 2000). Bacteroidetes in the human gut not only can effectively improve the degradation of dietary fiber, but also help host utilize dietary polysaccharide substance (Zhang, Chekan, et al., 2014; Zhang, Sun, et al., 2014). The gut microbiota in fish has major impact on growth, health, and development of fish (Dhanasiri et al., 2011; Ringø, Olsen, Mayhew, & Myklebust, 2003). Therefore, information on the composition of intestinal microbiota is important for intestinal development, homeostasis, and protection (Verschuere et al., 2000). Study on gut microbiota has been explored in many organisms and environments (Edwards et al., 2015; Peiffer et al., 2013; Rietl, Overlander, Nyman, & Jackson, 2016), but there is relatively little research into the gut microbiota of freshwater bivalves. Compared with terrestrial organisms, aquatic organisms inhabit in a more complex ecological environment, and their community structure of gut microbiota may have more variety and complexity (Nayak, 2010).

Many worldwide capture fisheries continue to decline and the human population continues to increase exponentially (Béné et al., 2015). Freshwater mussels are used for human and livestock food, and their shells are used for making buttons, shell inlay, beads, and pearls (Xiong, Ouyang, & Wu, 2012; Lopes‐Lima et al., 2017). For example, *S. carinata* is harvested for human consumption, having been found for sale in local markets from Poyang Lake (Liu & Wu, 1991; Sun et al., 2018). A large‐scale harvest for button manufacturing and pearl farming has persisted in the Yangtze River since the middle of the 19th century (Wu, Liang, Wang, Xie, & Ouyang, 2000; Xiong et al., 2012). This excessive exploitation and utilization lead to a serious decline in mussel populations (Liu, Yang, et al., 2020). Therefore, freshwater mussels are among endangered faunas worldwide (Bogan, 2008; Lopes‐Lima et al., 2017; Strayer et al., 2004). According to the IUCN Red List of Threatened Species, approximately 6% of known species have recently become extinct, and 40% have been identified as extinct, endangered, threatened, or near threatened (IUCN, 2019).

Freshwater mussels (Unionoida: Unionidae) are filter‐feeding bivalves that reside in sediment and consume bacteria, phytoplankton, detritus, and particulate organic matter in freshwater ecosystem (Vaughn, 2012, 2018). Freshwater mussels provide important ecosystem services, such as turning over sediments, filtering water, and maintaining its quality (Atkinson, Vaughn, Forshay, & Cooper, 2013; Vaughn, 2018). The gut microbiota in freshwater mussels has major impact on growth, health, and development of them (Aceves, Johnson, Bullard, Lafrentz, & Arias, 2018; Weingarten, Atkinson, & Jackson, 2019). Many studies on freshwater mussels have focused on feeding behavior (Vaughn, 2018) and diet (Atkinson et al., 2013; Christian, Smith, Berg, Smoot, & Findley, 2004; Vaughn & Hakenkamp, 2001), little is known about its diversity and functional role inside intestinal ecosystems of wild freshwater mussels. In order to promote the health of wild freshwater mussels, it is necessary to pay attention and study the community structure of gut microbiota and the factors affecting the composition and stability of them (Bahrndorff, Alemu, Alemneh, & Nielsen, 2016). Information of the gut microbiota could have significant implications for conservation biology, especially for threatened or endangered species (Bahrndorff et al., 2016). In an effort to describe the gut microbiota of wild freshwater mussel, and also to promote the study and understanding of microbial coevolution, the present study was to characterize the gut microbiota diversity of wild freshwater mussels. Here, the gut microbiota diversity was analyzed in endangered, economical, and common freshwater mussels using 16S rRNA gene sequencing. This study will provide important information for the conservation of freshwater mussels and may inform future studies on microbial ecology as well as other mussel health.

## 2 MATERIALS AND METHODS

### 2.1 Sample collection and DNA extraction

The specimens of *Sinohyriopsis cumingii* (code: SJ; economical freshwater mussels; 3 samples) and *Sinanodonta woodiana* (code: BJ; common freshwater mussels; 3 samples) were collected in the Gan River (28.68N, 115.86E), and *Solenaia carinata* (code: LG; endangered freshwater mussels; 3 samples) from the Fu River (28.52N, 116.10E). These mussels were cleaned with 70% alcohol and sterile water, obtaining the gut microbiota from the rectum. The gut microbiota was stored at‐80°C until DNA extraction. The genomic DNA was extracted from the gut microbiota of freshwater mussels using the TINAamp Marine Animals DNA Kit (TianGen). Concentration and quality of extracted DNA were estimated using a Nanodrop 2000 spectrophotometer (Thermo Scientific) and agarose gel electrophoresis.

### 2.2 PCR amplification

PCR amplification of the 16S rRNA genes V3–V4 region was performed using the forward primer 341F (5'‐CCTAYGGGRBGCASCAG‐3') and the reverse primer 806R (5'‐ GGACTACNNGGGTATCTAAT‐3'). Sample‐specific 8‐bp barcode barcodes were incorporated into the primers for multiplex sequencing. The polymerase chain reaction was carried out in a 20 µl volume containing 4 µL 5× FastPfu Buffer; 10 µl ddH_2_O; 0.8 µl of 5 µM forward primer; 0.8 µl of 5 µM reverse primer; 2 µl of 2.5 mMdNTPs; 0.4 µl FastPfu Polymerase; and 10 ng genomic DNA. PCR amplifications were conducted with the following touchdown thermal cycling program: an initial denaturation at 95°C for 5 min, followed by 27 cycles of 95°C for 30 s, annealing temperature of 55°C for 30 s, 72°C for 45 s, and a final extension at 72°C for 10 min.

### 2.3 Illumina MiSeq sequencing and statistical analysis

PCR amplification products were sequenced by the Illumina MiSeq platform from Mega Genomics Company (Degnan &and Ochman, 2012). The sequence was assembled by FLASH software (Magoc & Salzberg, 2011). Raw fastq files were demultiplexed and quality filtered using QIIME 1.17 (Caporaso et al., 2010) according to the following criteria: (a) the 250‐bp reads were truncated at any site receiving average Phred scores of <20 over a 10‐bp sliding window. Truncated reads that were shorter than 50 bp were discarded; (b) exact barcode matching, two nucleotide mismatches in primer matching and reads containing ambiguous characters were removed. (c) only sequences that overlapped more than 10 bp were assembled according to their overlap sequence. Reads that could not be assembled were discarded. Operational Taxonomic Units (OTUs) were clustered with a 97% similarity cutoff using Mothur software (Edgar, 2010; Quast et al., 2013), and chimeric sequences were identified and removed using UCHIME (Edgar, Haas, Clemente, Quince, & Knight, 2011). The phylogenetic affiliation of each 16S rRNA gene sequence was analyzed against the silva (SSU115)16S rRNA database (Wang, Garrity, Tiedje, & Cole, 2007).

Rarefaction curves are the statistical expectation for observed accumulation curves (Gotelli & Colwell, 2001), which make the comparison of the statistically expected species richness of each gut microbiota community at the same sampling effort or abundance (Moreno & Halffter, 2001). Rarefaction curves were generated by Mothur software (Edgar, 2010; Quast et al., 2013). Species rank/abundance plots describe communities of organisms based on the abundance of the gut microbiota community (Magurran, 2004). To estimate the diversity and richness of OTUs among samples, alpha diversity indices, such as Chao1 richness estimator, Shannon diversity index were calculated using the number of OTUs based on Mothur software (Edgar, 2010; Quast et al., 2013). Venn diagram was generated to visualize the shared and unique OTUs among samples or groups using R package “Venn Diagram,” based on the occurrence of OTUs across samples/groups regardless of their relative abundance (Zaura, Keijser, Huse, & Crielaard, 2009). The ANOSIM (Analysis of similarities) and MRPP (Multi‐response Permutation Procedures) were used to determine differences in gut microbiota communities. The ANOSIM and MRPP were generated using R version 2.13.1 (R Development Core Team, 2011) and the VEGAN packages. To examine the similarity among gut microbiota communities, heat map figures, beta diversity, and principal coordinate analysis (PCoA) were used based on the OTU information from each sample using R version 2.13.1 (R Development Core Team, 2011) and the VEGAN packages.

## 3 RESULTS

### 3.1 Composition of gut microbiota in three freshwater mussels

**FIGURE 1:**
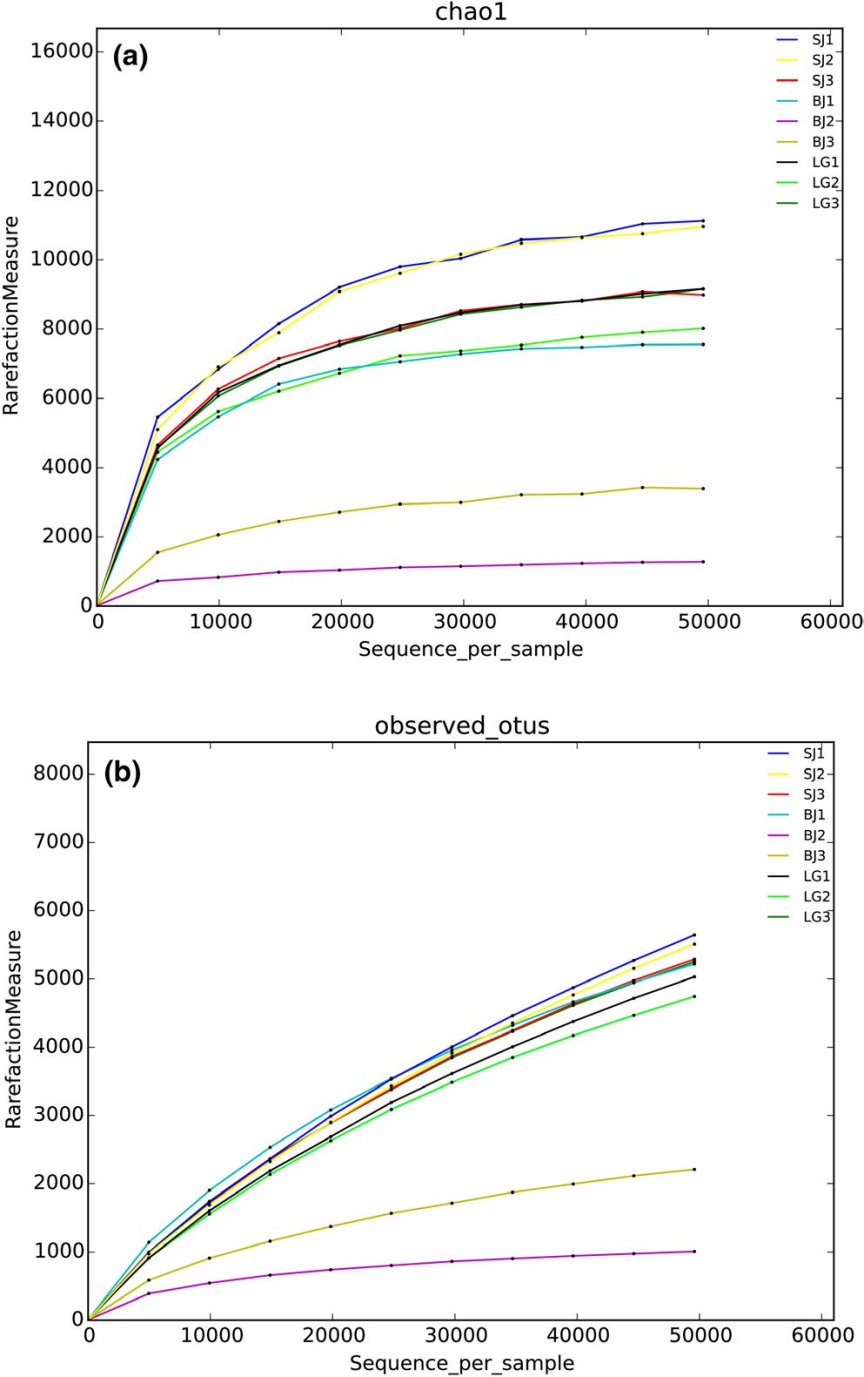
Chao1 rarefaction curves (a) and OTUs rarefaction curves (b) for relative abundance of gut microbiota from the mussel species *S. cumingii* (SJ), *S. woodiana* (BJ), and *S. carinata* (LG). Data were obtained using a threshold of 97%

**FIGURE 2:**
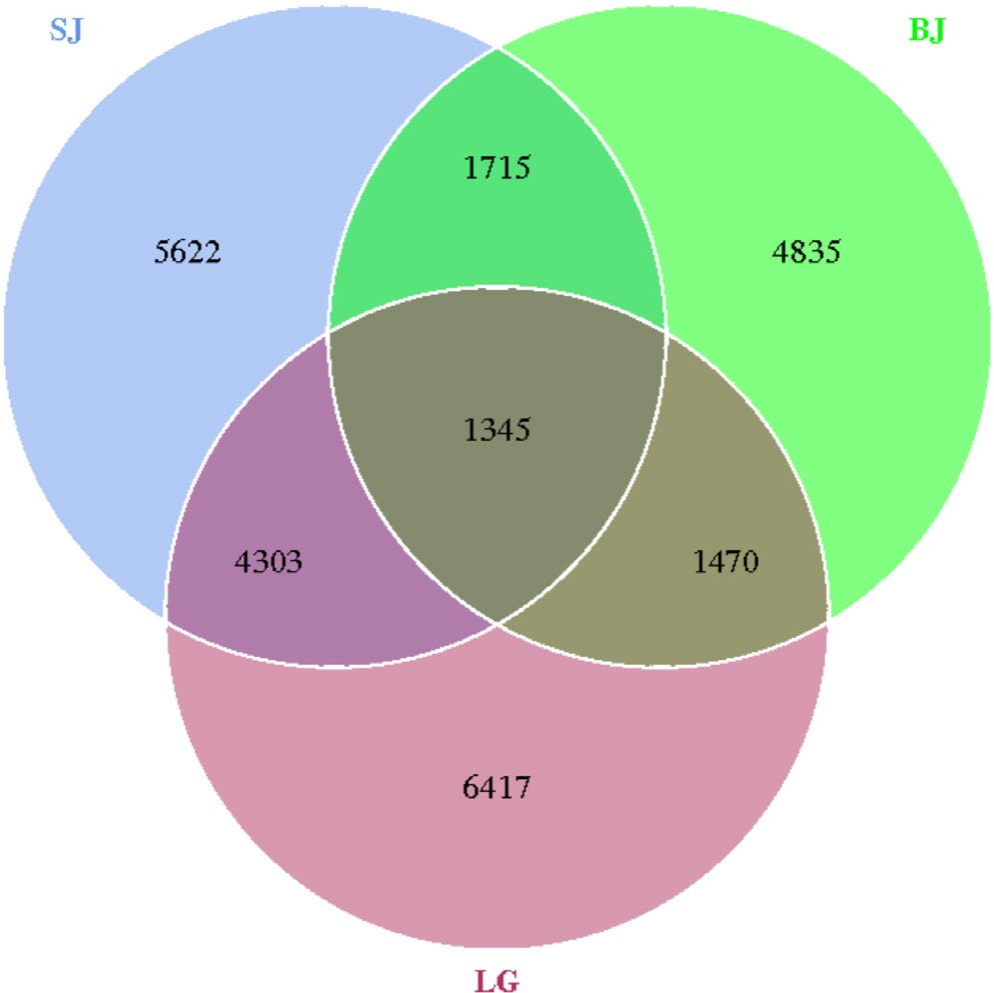
The Venn diagrams show the numbers of OTUs (97% sequence identity) that were shared or not shared gut microbiota among the mussel species *S. cumingii* (SJ), *S. woodiana* (BJ), and *S. carinata* (LG)

**FIGURE 3:**
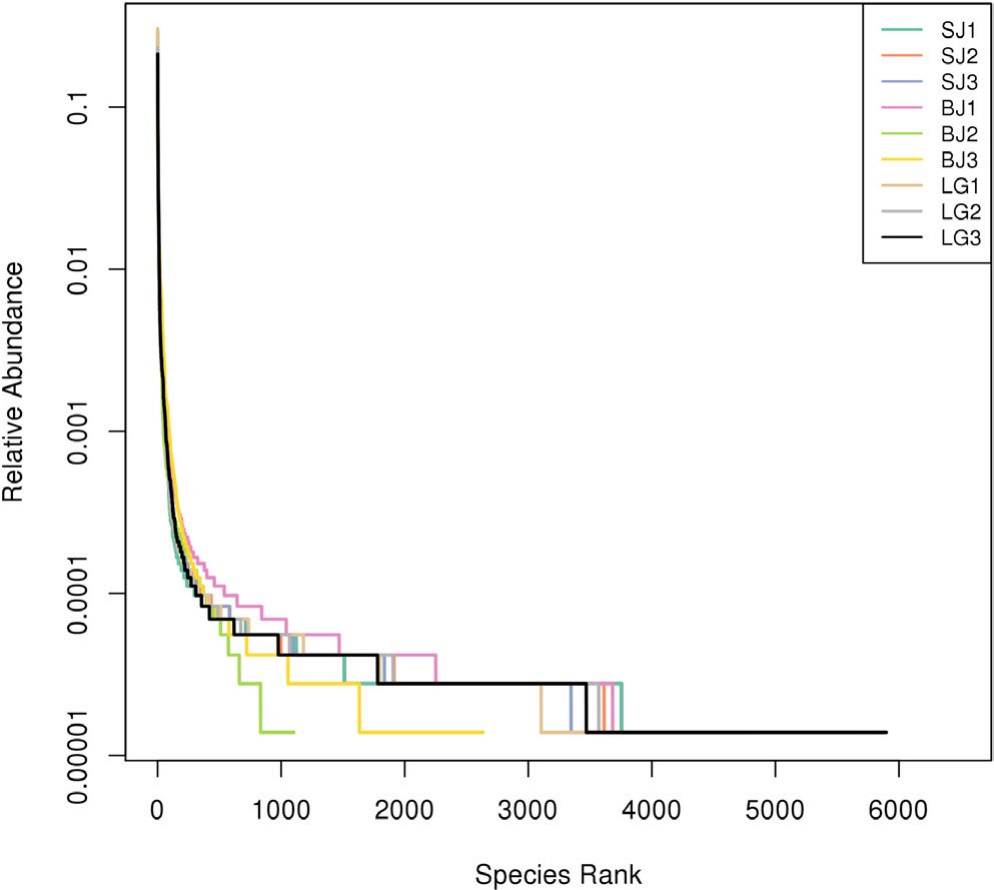
Rank‐abundance curves of gut microbiota present from the mussel species *S. cumingii* (SJ), *S. woodiana* (BJ), and *S. carinata* (LG). Data were obtained using a threshold of 97%

The amount of obtained sequences was sufficient to reasonably quantify the gut microbiota communities of three freshwater mussels because the sequences number of each sample OTU was distributed in the 97% sequence similarity threshold based on rarefaction curves (Figure 1).The 13,535 OTUs were found in *S. carinata*, 12,985 OTUs in *S. cumingii,* and 9,365 OTUs in *S. woodiana* (Table 1). The Venn diagrams showed that three freshwater mussels shared 1,345 OTUs, while 5,648 OTUs were shared between *S. carinata* and *S. cumingii*, 2,815 OTUs between *S. carinata* and *S. woodiana* and 3,060 OTUs between *S. carinata* and *S. woodiana* (Figure 2). The species rank curves showed that the bacterial relative abundance was very close to each other (Figure 3).

**TABLE 1:** Composition of gut microbiota in the mussel species *S. cumingii* (SJ), *S. woodiana* (BJ), and *S. carinata* (LG)

	LG	SJ	BJ
Sequences	60119–106882	73041–113684	69457–242659
Phylum	54	43	181
Class	281	390	273
Order	374	373	460
Family	8,235	7,718	2,733
Genus	3,993	4,124	4,717
OTUs	13,535	12,985	9,635

**FIGURE 4:**
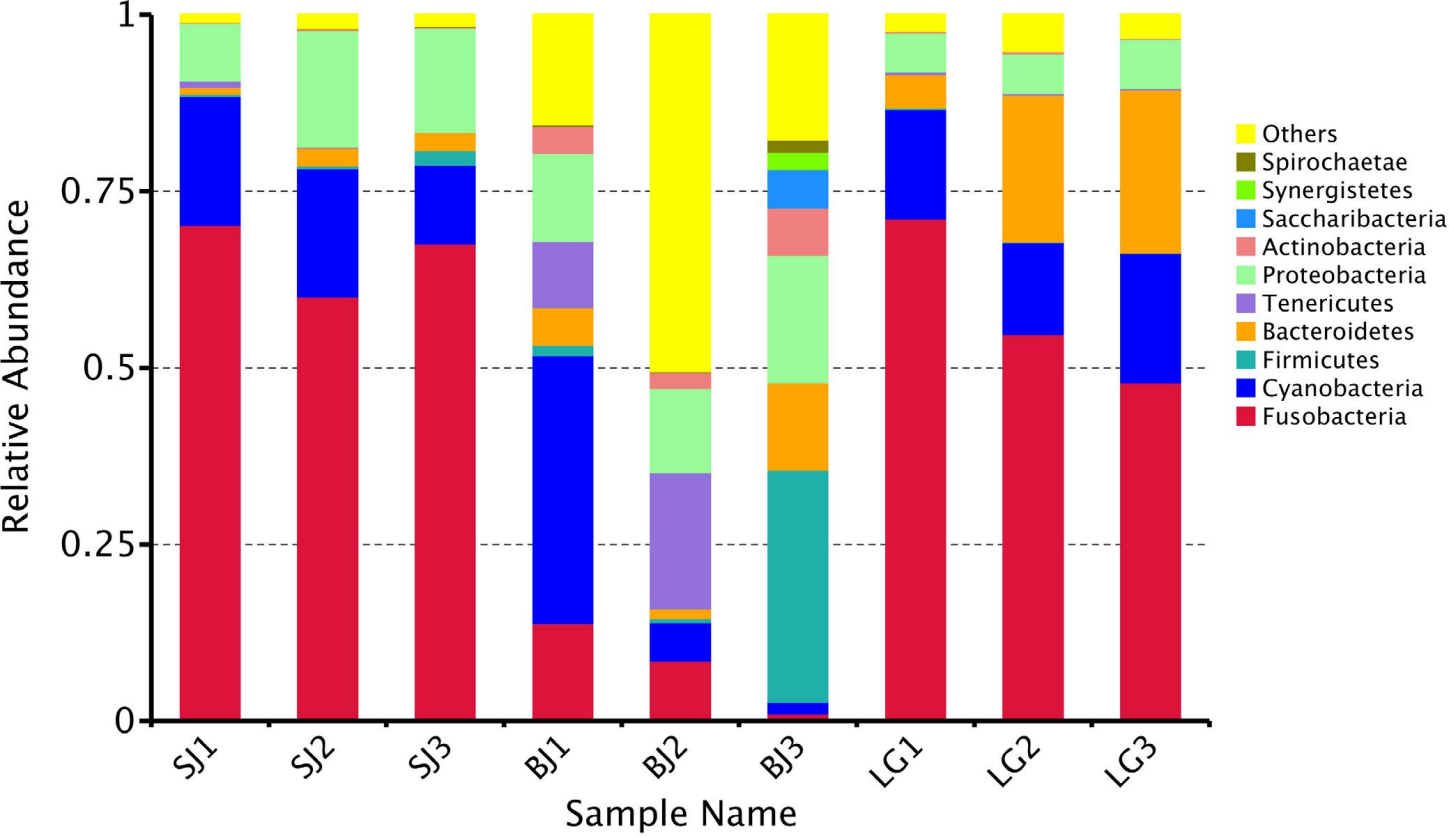
Relative abundance of gut microbiota from the mussel species *S. cumingii* (SJ), *S. woodiana* (BJ), and *S. carinata* (LG)

The detected gut microbiota in *S. carinata* and *S. cumingii* was classified into 54 and 43 phyla, 281 and 390 classes, 374 and 373 orders, 8,235 and 7,718 families, 3,993 and 4,124 genera, which dominant phylum was Fusobacteria, respectively (55.85% and 65.38%; Table 1, Figure 4).The detected gut microbiota in *S. woodiana* was classified into181 phyla, 273 classes, 460 orders, 2,733 families, and 4,717 genera, and dominant phylum was Firmicutes (21.84%).

### 3.2 Diversity of gut microbiota in three freshwater mussels

**FIGURE 5:**
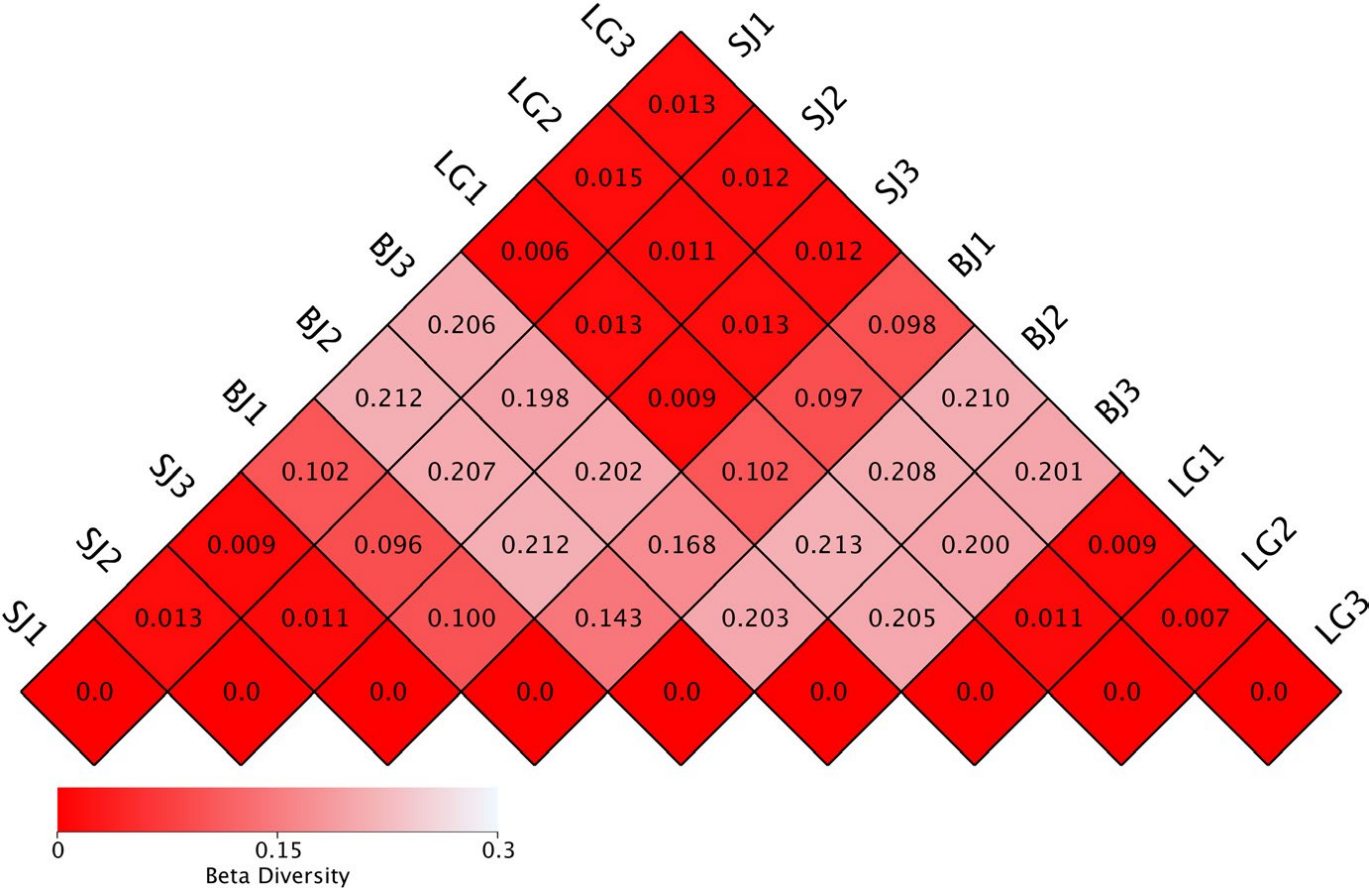
Analysis of beta diversity of gut microbiota from the mussel species *S. cumingii* (SJ), *S. woodiana* (BJ), and *S. carinata* (LG)

Alpha diversity indices of the gut microbiota showed that Chao1 index (9,798.9 and 7,969.1) and Shannon index (6.5 and 6.7) in *S. carinata* and *S. cumingii* were greater than in *S. woodiana* (1,123.7 and 4.8; Table 2). The value of beta diversity indices between *S. carinata* and *S. cumingii* was low, which indicated the composition of gut microbiota in *S. carinata* was similar with *S. cumingii*, while the composition of gut microbiota in *S. carinata* and *S. cumingii* was dissimilar with *S. woodiana* (Figure 5).

**TABLE 2:** Median (minimum‐maximum) alpha diversity indices of gut microbiota in the mussel species *S. cumingii* (SJ), *S. woodiana* (BJ), and *S. carinata* (LG)

	LG	SJ	BJ
Chao1	9,798.9 (15.1–11121.5)	7,969.1 (18.8–9151.9)	1,123.7 (15.1–7557.3)
OTUs	3,378.8 (7.4–5646.5)	3,541.8 (7.5–5287.7)	801.9 (7.2–5218)
Shannon	6.5 (2.7–6.8)	6.7 (2.7–7.5)	4.8 (2.7–6.7)

### 3.3 Community structure of gut microbiota in three freshwater mussels

**FIGURE 6:**
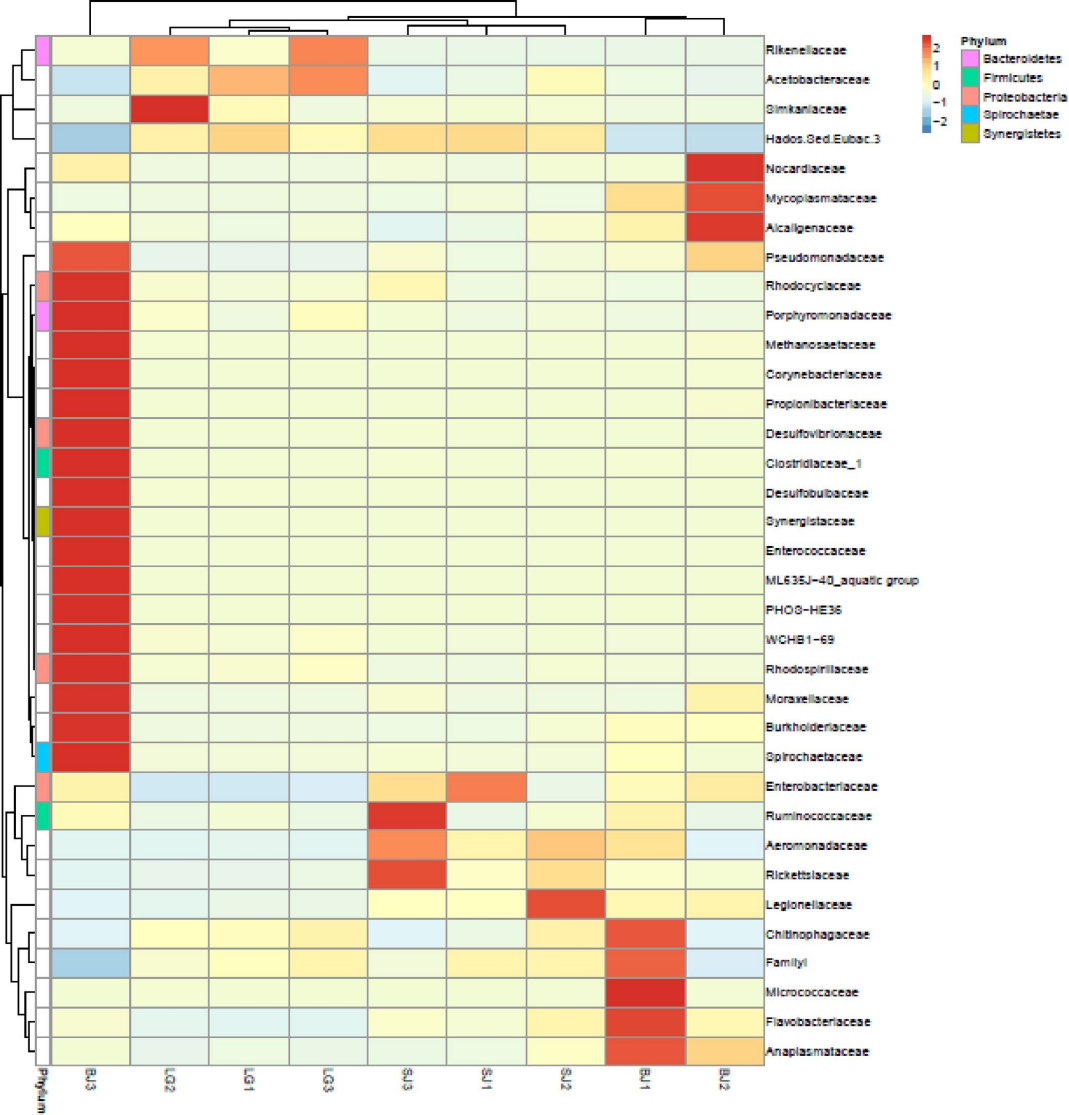
Heat map showing the clustering of relative abundances of the gut microbiota at the family level from the mussel species *S. cumingii* (SJ), *S. woodiana* (BJ), and *S. carinata* (LG)

A heat map analysis that the vertical clustering between *S. carinata* and *S. cumingii* showed a certain degree of similarity in richness (Figure 6).

**FIGURE 7:**
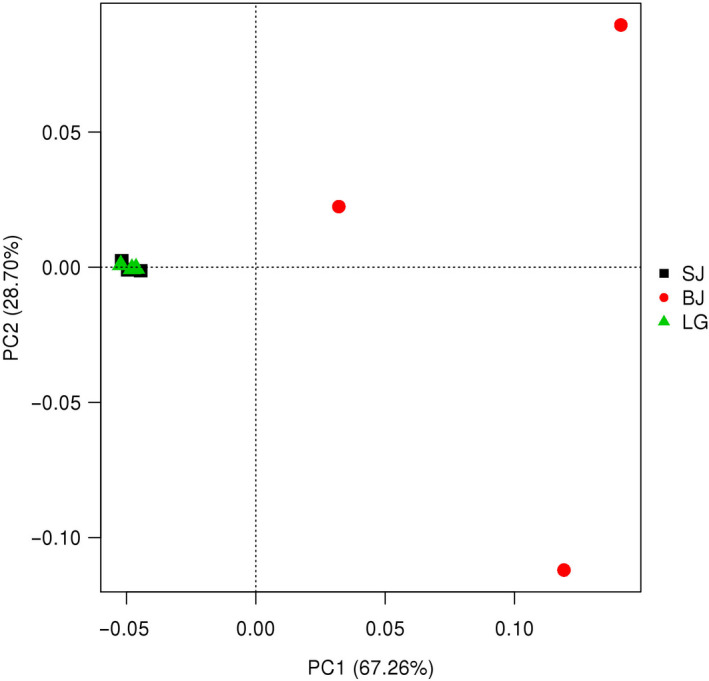
Principal coordinate analysis (PCoA) plots of 16S rRNA gut microbiota dataset collected from the mussel species *S. cumingii* (SJ), *S. woodiana* (BJ), and *S. carinata* (LG)

The PCoA showed that the assemblage structure of gut microbiota was divided into four groups with the first cluster being formed *S. carinata*, the second cluster formed *S. cumingii*, and three samples from *S. woodiana* were divided into two groups (Figure 7). In addition, the composition of gut microbiota between *S. carinata* and *S. cumingii* were similar, three samples from *S. woodiana* had a high variability along the axis (Figure 7).

The analysis of ANOSIM showed that the interspecific variation among three freshwater mussels was greater than the intraspecific variation (*R*＞0), but composition variation of gut microbiota among three freshwater mussels was not significant (*P *> .05; Table 3). The analysis of MRPP showed similar results to those resolved with ANOSIM analysis (*R *> 0; *P > *.05).

**TABLE 3:** Analysis of ANOSIM and MRPP of gut microbiota among the mussel species *S. cumingii* (SJ), *S. woodiana* (BJ), and *S. carinata* (LG)

	ANOSIM		MRPP			
	*R*	*p*	A	Observed delta	Expected delta	*p*
BJ‐LG	.63	.102	0.07	0.54	0.59	.10
SJ‐BJ	.56	.096	0.09	0.55	0.59	.10
SJ‐LG	.96	.079	0.06	0.46	0.49	.10

## 4 DISCUSSION

In this study, the dominant phylum in *S. carinata* and *S. cumingii* was Fusobacteria, and was Firmicutes in *S. woodiana*. Compared with freshwater mussels *Villosa nebulosa* from American, Tenericutes was the dominant phylum in all samples (>87%) using 16S rRNA gene pyrosequencing (Aceves et al., 2018). The dominant phylum in four freshwater mussels form North America were Proteobacteria and Firmicutes (Weingarten et al., 2019). Knowledge of gut microbiota of freshwater mussels can help for understanding how community structure is assembled and how they impact host fitness (Aceves et al., 2018; Weingarten et al., 2019). In addition, it is important for conservation of freshwater mussel biodiversity because of as filter feeders their gut microbiota may be particularly sensitive to environmental variation (Aceves et al., 2018; Vaughn, 2018; Weingarten et al., 2019). The factors that drive the community structure of gut microbiota have been analyzed for many species (Edwards et al., 2015; Peiffer et al., 2013; Rietl et al., 2016), but rarely so for freshwater mussels. For example, the dominant phylum in *Hypophthalmichthys molitrix*, *Megalobrama amblycephala* and *Oncorhynchus mykiss* was Proteobacteria and Firmicutes (Wong et al., 2013). The dominant phylum in *Penacus orientalis* was Firmicutes, Actinobacteria, and Fusobacteria (Rungrassamee et al., 2014; (Zhang, Chekan, et al., 2014; Zhang, Sun, et al., 2014). *C. virginica* were found to have gut microbiota dominated by members of the Pelagibacteraceae and genus *Synechococcus* (Ossai et al., 2017).

The composition of gut microbiota was affected by many factors, such as species, lifestyle, feeding habit, diet, nutritional status, and living conditions (Ley et al., 2008; Nayak, 2010; Schwab, Cristescu, Northrup, Stenhouse, & Ganzle, 2011). The different physicochemical conditions of the freshwater environment could generate different selection pressures for the recruitment of bacterial taxa (Weingarten et al., 2019). Some studies showed that the habitat characteristic of aquatic animals may be influence the composition of gut microbiota (Chauhan, Wafula, Lewis, & Pathak, 2014; Thomas et al., 2014). For example, the composition of gut microbiota has significant difference in marine and freshwater fish, and the salinity may one of factors influencing the composition of gut microbes (Sullam et al., 2012). While Weingarten et al. (2019) found that the structure of the gut microbiota of four co‐occurring freshwater mussels was differed in species or taxa composition, but were similar with marine system. Roeselers et al. (2011) found that the composition of gut microbiota in zebrafish was similar for different growth environment. In addition, feeding habits of aquatic animals may influence the composition of gut microbiota (Li, Yu, Feng, Yan, & Gong, 2012). For example, the gut microbiota of omnivorous *Carassius cuvieri* showed the higher diversity than those of carnivorous individuals, which means that feeding habits affected composition of gut microbiota (Ward, Steven, Penn, Methe, & Deteich, 2009). This study showed that the composition of gut microbiota of three freshwater mussels was different. *S. carinata* and *S. cumingii* are found in large river‐connected lakes, with relative rapid water flow, clear water, slightly hard sediment, and gravel substratum (Liu, Zhang, Wang, & Wang, 1979; Sun et al., 2018). *S. woodiana* has extensive habitat in lakes, rivers, reservoirs, and ponds with sediment or muddy substrate (Liu et al., 1979). Our study showed that the composition of gut microbiota between *S. carinata* and *S. cumingii* were similar.

The core gut microbiota of a species is defined as the group of microbes present in all individuals in different environment (Turnbaugh, Ley, Fraser‐Liggett, Knight, & Gordon, 2007). The core gut microbiota of freshwater mussels is not only to improve survivorship, but eventually to identify “normal” or “healthy” species (Aceves et al., 2018). The core gut microbiota could evaluate mussel mortality during kill events or disease epizooties (Southwick & Loftus, 2003). In our study, the Venn diagrams showed that three freshwater mussels were shared 1,345 OTUs, while 5,648 OTUs were shared in *S. carinata* and *S. cumingii*, 2,815 OTUs in *S. carinata* and *S. woodiana* and 3,060 OTUs in *S. carinata* and *S. woodiana*, suggesting that a core gut microbiota may exist among these species.

This study represents the first to compare the gut microbiota diversity in endangered, economical, and common Chinese freshwater mussels using 16S rRNA gene sequencing. The dominant phylum in *S. carinata* and *S. cumingii* was Fusobacteria, and was Firmicutes in *S. woodiana*. The composition of gut microbiota among three freshwater mussels was different, but their composition variation was not significant. The study aim of gut microbiota in freshwater mussels is not only analyzed their composition, but also need to reveal the role of gut microbiota in the host's nutrition metabolism or immune regulation. In order to further analyze gut microbiota of freshwater mussels, (a) it will require a much more powerful whole‐genome sequencing methods; (b) trying to separate functional microbiota from their gut based on composition of gut microbiota in freshwater mussels; (c) screening functional genes of gut microbiota based on metagenome sequencing; (d) study on the factors that drive the community structure of gut microbiota to realize the artificial regulation of the structure of gut microbiota.

## CONFLICT OF INTEREST

None declared.

## AUTHOR CONTRIBUTION

**Xiongjun Liu:** Conceptualization (equal); Data curation (equal); Formal analysis (equal); Methodology (equal); Writing‐original draft (equal); Writing‐review & editing (equal). **Yanling Cao:** Conceptualization (equal); Data curation (equal); Formal analysis (equal); Writing‐original draft (equal); Writing‐review & editing (equal). **Shan Ouyang:** Conceptualization (equal); Data curation (equal); Formal analysis (equal); Funding acquisition (equal); Project administration (equal); Writing‐original draft (equal); Writing‐review & editing (equal). **Xiaoping Wu:** Conceptualization (equal); Data curation (equal); Formal analysis (equal); Funding acquisition (equal); Project administration (equal); Writing‐original draft (equal); Writing‐review & editing (equal).

## Data Availability

All raw sequences were deposited in the NCBI Sequence Read Archive under accession number SRA Accession no. PRJNA322397.
